# Supplementary material for: Medicinal Plant Preparations Administered by Botswana Traditional Health Practitioners for Treatment of Worm Infections Show Anthelmintic Activities
Source: Plants (Basel). 2022 Nov 1;11(21):2945. doi: 10.3390/plants11212945 (PMC9658373; doi:10.3390/plants11212945)
Supplement: Supplementary file 1 [file plants-11-02945-s001.zip › plants-1931185-supplementary.pdf]

Supplementary Materials

Medicinal Plant Preparations Administered by Botswana Traditional Health Practitioners for Treatment of Worm Infections Show Anthelmintic Activities

Mthandazo Dube [1], Boingotlo Raphane [2], Bongani Sethebe [2], Nkaelang Seputhe6, Tsholofelo Tiroyakgosi6, Peter Imming [3], Cécile Häberli [4,5], Jennifer Keiser [4,5], Norbert Arnold [1], Kerstin Andrae-Marobela [2,*]

1 Department of Bioorganic Chemistry, Leibniz Institute of Plant Biochemistry, Weinberg 3, D-06120 Halle (Saale), Germany
2 Department of Biological Sciences, Faculty of Science, University of Botswana, P.O. Box 0022, Gaborone, Botswana
3 Institute of Pharmacy, Faculty of Natural Sciences, Martin-Luther-University Halle-Wittenberg, D-06120 Halle (Saale), Germany
4 Swiss Tropical and Public Health Institute, Socinstr. 57, CH-4051 Basel, Switzerland
5 University of Basel, CH-4051 Basel, Switzerland
6 Kwame Legwame Traditional Association, Maun, Botswana
* Correspondence: marobelak@ub.ac.bw; Tel.: +267 355 2609 (K.A.-M.)

Table S1: Anthelmintic activity against parasitic helminths.

	A. ferox leaves	*A. zebrina* leaves	*B. albitrunca* leaves	*C. mopane* seeds	*M. oleifera* leaves	*S. panduriforme* fruit	*T. sericea* roots
Organism	**Mortality rate %***						
NTS** (100 µg/mL)	46.15 ± 3.8	34.62 ± 3.8	86.54 ± 5.8	100 ± 0	90.38 ± 5.8	69.23 ± 3.8	86.54 ± 9.6
NTS** (50 µg/mL)	30.77 ± 0	30.77 ± 0	42.31 ± 3.8	55.77 ± 1.9	30.77 ± 0	61.54 ± 0	34.62 ± 3.8
A. ceylanicum L3 (100 µg/mL)	36.8 ± 7.1	29.2 ± 0.2	33.6 ± 2.3	54.5 ± 1.9	37.6 ± 1.9	19.4 ± 4.2	38.3 ± 7.8
H. polygyrus adult (100 µg/mL)	48.35 ± 8.3	31.65 ± 1.7	50 ± 6.7	53.35 ± 3.3	56.65 ± 3.3	56.65 ± 3.3	55 ± 1.7
H. polygyrus L3 (100 µg/mL)	7.9 ± 2.6	7.1 ± 3.3	23.2 ± 2	11.1 ± 4.4	14.3 ± 1.6	10.3 ± 0.1	14.4 ± 0.5
N. americanus L3 (100 µg/mL)	11.8 ± 4.8	3.5 ± 2.7	0.3 ± 1.5	4.3 ± 3.6	6.5 ± 5	15.3 ± 5.1	15.4 ± 2.7
S. ratti L3 (100 µg/mL)	51.8 ± 4.6	28.4 ± 4.7	36.4 ± 3.4	21.9 ± 6.8	58.2 ± 9.8	34.4 ± 2.1	36.8 ± 2.3
T. muris adult (100 µg/mL)	33.35 ± 3.3	38.35 ± 1.7	38.35 ± 1.7	55.5 ± 5	40 ± 0	66.65 ± 3.3	40 ± 0
T. muris adult (50 µg/mL)	nt	nt	nt	nt	nt	29.6 ± 0	nt

*mortality % based on three replicates; ** NTS = newly transformed schistosomula; nt = not tested

Table S2: Most active plants against *S. mansoni* and NTS.

	L. luteoalbum roots	*L. luteoalbum* leaves	*C. pyracanthoides* stem bark	*C. imberbe* leaves
**Organism**	**Mortality rate %***			
NTS** (100 µg/mL)	96.15 ± 3.8	96.15 ± 3.8	80.77 ± 3.8	100 ± 0
NTS** (50 µg/mL)	90.38 ± 1.9	94.32 ± 1.9	76.92 ± 0	98.08 ± 1.9
NTS** (10 µg/mL)	87.5 ± 0	91.67 ± 0	52.08 ± 2.1	29.17 ± 4.2
NTS** (1 µg/mL)	85.42 ± 2.1	85.42 ± 2.1	nt	nt
NTS** (0.1 µg/mL)	35.71 ± 0	28.57 ± 3.6	nt	nt
S. mansoni adults (50 µg/mL)	46.95 ± 2	67.65 ± 3.9	100 ± 0	78.4 ± 5.9
S. mansoni adults (10 µg/mL)	32.05 ± 3.6	37.4 ± 1.8	78.5 ± 0	32.05 ± 3.6
S. mansoni adults (1 µg/mL)	nt	nt	41.65 ± 1.7	nt
A. ceylanicum L3 (100 µg/mL)	45 ± 7.5	53.1 ± 5.2	63.3 ± 6.9	45.6 ± 0.8
H. polygyrus adult (100 µg/mL)	51.65 ± 1.7	46.7 ± 0	43.35 ± 3.3	40 ± 0
H. polygyrus L3 (100 µg/mL)	14.8 ± 0.7	11.3 ± 0.6	18.5 ± 3.9	7.5 ± 3.4
N. americanus L3 (100 µg/mL)	6.9 ± 4	16.5 ± 9.7	22.2 ± 2.7	7.5 ± 4.9
S. ratti L3 (100 µg/mL)	40.2 ± 4.5	7 ± 7.4	39.2 ± 9.9	11 ± 5.6
T. muris adult (100 µg/mL)	40 ± 0	41.65 ± 1.7	58.35 ± 1.7	20 ± 0

*mortality % based on three replicates; ** NTS = newly transformed schistosomula; nt = not tested

Table S3: **Statistical tables for one way ANOVA using Sigma Plot 14.0. ANOVA table for data in figure 1.**

**Source of Variation**	**DF**	**SS**	**MS**	**F**	**P**
Between Groups	87	177455,140	2039,714	120,420	<0,001
Residual	176	2981,140	16,938		
Total	263	180436,280			

The differences in the mean values among the treatment groups are greater than would be expected by chance; there is a statistically significant difference (P = <0,001).

All Pairwise Multiple Comparison Procedures (Tukey Test):

Comparisons for factor:

**Comparison**	**Diff of Means**	**p**	**q**	**P**	**P<0,050**
Row 33 vs. Row 22	99,700	88	41,959	<0,001	Yes
Row 33 vs. Row 14	96,500	88	40,612	<0,001	Yes
Row 33 vs. Row 30	95,700	88	40,275	<0,001	Yes
Row 33 vs. Row 53	93,500	88	39,349	<0,001	Yes
Row 33 vs. Row 78	93,100	88	39,181	<0,001	Yes
Row 33 vs. Row 87	93,000	88	39,139	<0,001	Yes
Row 33 vs. Row 13	92,900	88	39,097	<0,001	Yes
Row 33 vs. Row 37	92,500	88	38,928	<0,001	Yes

Row 33 vs. Row 5	92,100	8838,760	<0,001	Yes
Row 33 vs. Row 60	89,700	8837,750	<0,001	Yes
Row 33 vs. Row 38	89,000	8837,456	<0,001	Yes
Row 33 vs. Row 29	88,900	8837,413	<0,001	Yes
Row 33 vs. Row 85	88,700	8837,329	<0,001	Yes
Row 33 vs. Row 6	88,200	8837,119	<0,001	Yes
Row 33 vs. Row 52	85,700	8836,067	<0,001	Yes
Row 33 vs. Row 69	85,600	8836,025	<0,001	Yes
Row 33 vs. Row 77	85,200	8835,856	<0,001	Yes
Row 33 vs. Row 61	84,700	8835,646	<0,001	Yes
Row 33 vs. Row 70	84,600	8835,604	<0,001	Yes
Row 33 vs. Row 86	83,500	8835,141	<0,001	Yes
Row 33 vs. Row 44	81,500	8834,299	<0,001	Yes
Row 33 vs. Row 58	80,600	8833,920	<0,001	Yes
Row 33 vs. Row 39	80,000	8833,668	<0,001	Yes
Row 33 vs. Row 31	78,100	8832,868	<0,001	Yes
Row 33 vs. Row 45	77,800	8832,742	<0,001	Yes
Row 33 vs. Row 21	76,800	8832,321	<0,001	Yes
Row 33 vs. Row 15	71,600	8830,133	<0,001	Yes
Row 33 vs. Row 11	70,800	8829,796	<0,001	Yes
Row 33 vs. Row 64	70,400	8829,628	<0,001	Yes
Row 33 vs. Row 10	69,230	8829,135	<0,001	Yes
Row 33 vs. Row 49	69,230	8829,135	<0,001	Yes
Row 33 vs. Row 2	69,230	8829,135	<0,001	Yes
Row 33 vs. Row 12	68,350	8828,765	<0,001	Yes
Row 33 vs. Row 8	66,650	8828,050	<0,001	Yes
Row 33 vs. Row 19	66,400	8827,944	<0,001	Yes
Row 33 vs. Row 62	65,600	8827,608	<0,001	Yes
Row 33 vs. Row 9	65,380	8827,515	<0,001	Yes
Row 33 vs. Row 66	65,380	8827,515	<0,001	Yes
Row 33 vs. Row 23	63,600	8826,766	<0,001	Yes
Row 33 vs. Row 3	63,200	8826,598	<0,001	Yes
Row 33 vs. Row 71	63,200	8826,598	<0,001	Yes
Row 33 vs. Row 50	62,400	8826,261	<0,001	Yes
Row 33 vs. Row 67	61,700	8825,966	<0,001	Yes
Row 33 vs. Row 16	61,650	8825,945	<0,001	Yes
Row 33 vs. Row 24	61,650	8825,945	<0,001	Yes
Row 33 vs. Row 46	60,800	8825,588	<0,001	Yes
Row 33 vs. Row 55	60,000	8825,251	<0,001	Yes
Row 33 vs. Row 36	60,000	8825,251	<0,001	Yes
Row 33 vs. Row 72	60,000	8825,251	<0,001	Yes
Row 33 vs. Row 80	60,000	8825,251	<0,001	Yes
Row 33 vs. Row 79	59,800	8825,167	<0,001	Yes
Row 33 vs. Row 88	58,350	8824,557	<0,001	Yes
Row 33 vs. Row 18	57,690	8824,279	<0,001	Yes
Row 33 vs. Row 43	56,650	8823,841	<0,001	Yes
Row 33 vs. Row 75	55,000	8823,147	<0,001	Yes
Row 33 vs. Row 35	54,400	8822,894	<0,001	Yes
Row 33 vs. Row 1	53,850	8822,663	<0,001	Yes
Row 33 vs. Row 84	53,300	8822,431	<0,001	Yes
Row 33 vs. Row 4	51,650	8821,737	<0,001	Yes
Row 33 vs. Row 20	50,000	8821,042	<0,001	Yes
Row 33 vs. Row 76	48,350	8820,348	<0,001	Yes
Row 33 vs. Row 7	48,200	8820,285	<0,001	Yes
Row 33 vs. Row 83	46,900	8819,738	<0,001	Yes
Row 33 vs. Row 28	46,650	8819,633	<0,001	Yes
Row 33 vs. Row 27	45,500	8819,149	<0,001	Yes
Row 33 vs. Row 68	45,000	8818,938	<0,001	Yes
Row 33 vs. Row 32	44,500	8818,728	<0,001	Yes
Row 33 vs. Row 26	44,230	8818,614	<0,001	Yes
Row 33 vs. Row 59	43,350	8818,244	<0,001	Yes
Row 33 vs. Row 51	43,350	8818,244	<0,001	Yes
Row 33 vs. Row 54	41,800	8817,591	<0,001	Yes
Row 33 vs. Row 47	41,650	8817,528	<0,001	Yes
Row 33 vs. Row 57	38,460	8816,186	<0,001	Yes
Row 33 vs. Row 56	37,070	8815,601	<0,001	Yes

Row 33 vs. Row 42	36,700	8815,445	<0,001	Yes
Row 33 vs. Row 63	33,350	8814,035	<0,001	Yes
Row 33 vs. Row 41	23,080	88 9,713	<0,001	Yes
Row 33 vs. Row 40	19,230	88 8,093	<0,001	Yes
Row 33 vs. Row 65	13,460	88 5,665	0,117	No
Row 33 vs. Row 17	13,460	88 5,665	0,117	Do Not Test
Row 33 vs. Row 74	9,620	88 4,049	0,939	Do Not Test
Row 33 vs. Row 48	9,620	88 4,049	0,939	Do Not Test
Row 33 vs. Row 82	5,680	88 2,390	1,000	Do Not Test
Row 33 vs. Row 73	3,850	88 1,620	1,000	Do Not Test
Row 33 vs. Row 81	3,850	88 1,620	1,000	Do Not Test
Row 33 vs. Row 34	1,920	88 0,808	1,000	Do Not Test
Row 33 vs. Row 25	0,000	88 0,000	1,000	Do Not Test
Row 25 vs. Row 22	99,700	8841,959	<0,001	Yes
Row 25 vs. Row 14	96,500	8840,612	<0,001	Yes
Row 25 vs. Row 30	95,700	8840,275	<0,001	Yes
Row 25 vs. Row 53	93,500	8839,349	<0,001	Yes
Row 25 vs. Row 78	93,100	8839,181	<0,001	Yes
Row 25 vs. Row 87	93,000	8839,139	<0,001	Yes
Row 25 vs. Row 13	92,900	8839,097	<0,001	Yes
Row 25 vs. Row 37	92,500	8838,928	<0,001	Yes
Row 25 vs. Row 5	92,100	8838,760	<0,001	Yes
Row 25 vs. Row 60	89,700	8837,750	<0,001	Yes
Row 25 vs. Row 38	89,000	8837,456	<0,001	Yes
Row 25 vs. Row 29	88,900	8837,413	<0,001	Yes
Row 25 vs. Row 85	88,700	8837,329	<0,001	Yes
Row 25 vs. Row 6	88,200	8837,119	<0,001	Yes
Row 25 vs. Row 52	85,700	8836,067	<0,001	Yes
Row 25 vs. Row 69	85,600	8836,025	<0,001	Yes
Row 25 vs. Row 77	85,200	8835,856	<0,001	Yes
Row 25 vs. Row 61	84,700	8835,646	<0,001	Yes
Row 25 vs. Row 70	84,600	8835,604	<0,001	Yes
Row 25 vs. Row 86	83,500	8835,141	<0,001	Yes
Row 25 vs. Row 44	81,500	8834,299	<0,001	Yes
Row 25 vs. Row 58	80,600	8833,920	<0,001	Yes
Row 25 vs. Row 39	80,000	8833,668	<0,001	Yes
Row 25 vs. Row 31	78,100	8832,868	<0,001	Yes
Row 25 vs. Row 45	77,800	8832,742	<0,001	Yes
Row 25 vs. Row 21	76,800	8832,321	<0,001	Yes
Row 25 vs. Row 15	71,600	8830,133	<0,001	Yes
Row 25 vs. Row 11	70,800	8829,796	<0,001	Yes
Row 25 vs. Row 64	70,400	8829,628	<0,001	Yes
Row 25 vs. Row 10	69,230	8829,135	<0,001	Yes
Row 25 vs. Row 49	69,230	8829,135	<0,001	Yes
Row 25 vs. Row 2	69,230	8829,135	<0,001	Yes
Row 25 vs. Row 12	68,350	8828,765	<0,001	Yes
Row 25 vs. Row 8	66,650	8828,050	<0,001	Yes
Row 25 vs. Row 19	66,400	8827,944	<0,001	Yes
Row 25 vs. Row 62	65,600	8827,608	<0,001	Yes
Row 25 vs. Row 9	65,380	8827,515	<0,001	Yes
Row 25 vs. Row 66	65,380	8827,515	<0,001	Yes
Row 25 vs. Row 23	63,600	8826,766	<0,001	Yes
Row 25 vs. Row 3	63,200	8826,598	<0,001	Yes
Row 25 vs. Row 71	63,200	8826,598	<0,001	Yes
Row 25 vs. Row 50	62,400	8826,261	<0,001	Yes
Row 25 vs. Row 67	61,700	8825,966	<0,001	Yes
Row 25 vs. Row 16	61,650	8825,945	<0,001	Yes
Row 25 vs. Row 24	61,650	8825,945	<0,001	Yes
Row 25 vs. Row 46	60,800	8825,588	<0,001	Yes
Row 25 vs. Row 55	60,000	8825,251	<0,001	Yes
Row 25 vs. Row 36	60,000	8825,251	<0,001	Yes
Row 25 vs. Row 72	60,000	8825,251	<0,001	Yes
Row 25 vs. Row 80	60,000	8825,251	<0,001	Yes
Row 25 vs. Row 79	59,800	8825,167	<0,001	Yes
Row 25 vs. Row 88	58,350	8824,557	<0,001	Yes
Row 25 vs. Row 18	57,690	8824,279	<0,001	Yes

Row 25 vs. Row 43	56,650	8823,841	<0,001	Yes
Row 25 vs. Row 75	55,000	8823,147	<0,001	Yes
Row 25 vs. Row 35	54,400	8822,894	<0,001	Yes
Row 25 vs. Row 1	53,850	8822,663	<0,001	Yes
Row 25 vs. Row 84	53,300	8822,431	<0,001	Yes
Row 25 vs. Row 4	51,650	8821,737	<0,001	Yes
Row 25 vs. Row 20	50,000	8821,042	<0,001	Yes
Row 25 vs. Row 76	48,350	8820,348	<0,001	Yes
Row 25 vs. Row 7	48,200	8820,285	<0,001	Yes
Row 25 vs. Row 83	46,900	8819,738	<0,001	Yes
Row 25 vs. Row 28	46,650	8819,633	<0,001	Yes
Row 25 vs. Row 27	45,500	8819,149	<0,001	Yes
Row 25 vs. Row 68	45,000	8818,938	<0,001	Yes
Row 25 vs. Row 32	44,500	8818,728	<0,001	Yes
Row 25 vs. Row 26	44,230	8818,614	<0,001	Yes
Row 25 vs. Row 59	43,350	8818,244	<0,001	Yes
Row 25 vs. Row 51	43,350	8818,244	<0,001	Yes
Row 25 vs. Row 54	41,800	8817,591	<0,001	Yes
Row 25 vs. Row 47	41,650	8817,528	<0,001	Yes
Row 25 vs. Row 57	38,460	8816,186	<0,001	Yes
Row 25 vs. Row 56	37,070	8815,601	<0,001	Yes
Row 25 vs. Row 42	36,700	8815,445	<0,001	Yes
Row 25 vs. Row 63	33,350	8814,035	<0,001	Yes
Row 25 vs. Row 41	23,080	88 9,713	<0,001	Yes
Row 25 vs. Row 40	19,230	88 8,093	<0,001	Yes
Row 25 vs. Row 65	13,460	88 5,665	0,117	Do Not Test
Row 25 vs. Row 17	13,460	88 5,665	0,117	Do Not Test
Row 25 vs. Row 74	9,620	88 4,049	0,939	Do Not Test
Row 25 vs. Row 48	9,620	88 4,049	0,939	Do Not Test
Row 25 vs. Row 82	5,680	88 2,390	1,000	Do Not Test
Row 25 vs. Row 73	3,850	88 1,620	1,000	Do Not Test
Row 25 vs. Row 81	3,850	88 1,620	1,000	Do Not Test
Row 25 vs. Row 34	1,920	88 0,808	1,000	Do Not Test
Row 34 vs. Row 22	97,780	8841,151	<0,001	Yes
Row 34 vs. Row 14	94,580	8839,804	<0,001	Yes
Row 34 vs. Row 30	93,780	8839,467	<0,001	Yes
Row 34 vs. Row 53	91,580	8838,541	<0,001	Yes
Row 34 vs. Row 78	91,180	8838,373	<0,001	Yes
Row 34 vs. Row 87	91,080	8838,331	<0,001	Yes
Row 34 vs. Row 13	90,980	8838,289	<0,001	Yes
Row 34 vs. Row 37	90,580	8838,120	<0,001	Yes
Row 34 vs. Row 5	90,180	8837,952	<0,001	Yes
Row 34 vs. Row 60	87,780	8836,942	<0,001	Yes
Row 34 vs. Row 38	87,080	8836,647	<0,001	Yes
Row 34 vs. Row 29	86,980	8836,605	<0,001	Yes
Row 34 vs. Row 85	86,780	8836,521	<0,001	Yes
Row 34 vs. Row 6	86,280	8836,311	<0,001	Yes
Row 34 vs. Row 52	83,780	8835,259	<0,001	Yes
Row 34 vs. Row 69	83,680	8835,217	<0,001	Yes
Row 34 vs. Row 77	83,280	8835,048	<0,001	Yes
Row 34 vs. Row 61	82,780	8834,838	<0,001	Yes
Row 34 vs. Row 70	82,680	8834,796	<0,001	Yes
Row 34 vs. Row 86	81,580	8834,333	<0,001	Yes
Row 34 vs. Row 44	79,580	8833,491	<0,001	Yes
Row 34 vs. Row 58	78,680	8833,112	<0,001	Yes
Row 34 vs. Row 39	78,080	8832,860	<0,001	Yes
Row 34 vs. Row 31	76,180	8832,060	<0,001	Yes
Row 34 vs. Row 45	75,880	8831,934	<0,001	Yes
Row 34 vs. Row 21	74,880	8831,513	<0,001	Yes
Row 34 vs. Row 15	69,680	8829,325	<0,001	Yes
Row 34 vs. Row 11	68,880	8828,988	<0,001	Yes
Row 34 vs. Row 64	68,480	8828,820	<0,001	Yes
Row 34 vs. Row 10	67,310	8828,327	<0,001	Yes
Row 34 vs. Row 49	67,310	8828,327	<0,001	Yes
Row 34 vs. Row 2	67,310	8828,327	<0,001	Yes
Row 34 vs. Row 12	66,430	8827,957	<0,001	Yes

Row 34 vs. Row 8	64,730	8827,242	<0,001	Yes
Row 34 vs. Row 19	64,480	8827,136	<0,001	Yes
Row 34 vs. Row 62	63,680	8826,800	<0,001	Yes
Row 34 vs. Row 9	63,460	8826,707	<0,001	Yes
Row 34 vs. Row 66	63,460	8826,707	<0,001	Yes
Row 34 vs. Row 23	61,680	8825,958	<0,001	Yes
Row 34 vs. Row 3	61,280	8825,790	<0,001	Yes
Row 34 vs. Row 71	61,280	8825,790	<0,001	Yes
Row 34 vs. Row 50	60,480	8825,453	<0,001	Yes
Row 34 vs. Row 67	59,780	8825,158	<0,001	Yes
Row 34 vs. Row 16	59,730	8825,137	<0,001	Yes
Row 34 vs. Row 24	59,730	8825,137	<0,001	Yes
Row 34 vs. Row 46	58,880	8824,780	<0,001	Yes
Row 34 vs. Row 55	58,080	8824,443	<0,001	Yes
Row 34 vs. Row 36	58,080	8824,443	<0,001	Yes
Row 34 vs. Row 72	58,080	8824,443	<0,001	Yes
Row 34 vs. Row 80	58,080	8824,443	<0,001	Yes
Row 34 vs. Row 79	57,880	8824,359	<0,001	Yes
Row 34 vs. Row 88	56,430	8823,748	<0,001	Yes
Row 34 vs. Row 18	55,770	8823,471	<0,001	Yes
Row 34 vs. Row 43	54,730	8823,033	<0,001	Yes
Row 34 vs. Row 75	53,080	8822,339	<0,001	Yes
Row 34 vs. Row 35	52,480	8822,086	<0,001	Yes
Row 34 vs. Row 1	51,930	8821,855	<0,001	Yes
Row 34 vs. Row 84	51,380	8821,623	<0,001	Yes
Row 34 vs. Row 4	49,730	8820,929	<0,001	Yes
Row 34 vs. Row 20	48,080	8820,234	<0,001	Yes
Row 34 vs. Row 76	46,430	8819,540	<0,001	Yes
Row 34 vs. Row 7	46,280	8819,477	<0,001	Yes
Row 34 vs. Row 83	44,980	8818,930	<0,001	Yes
Row 34 vs. Row 28	44,730	8818,825	<0,001	Yes
Row 34 vs. Row 27	43,580	8818,341	<0,001	Yes
Row 34 vs. Row 68	43,080	8818,130	<0,001	Yes
Row 34 vs. Row 32	42,580	8817,920	<0,001	Yes
Row 34 vs. Row 26	42,310	8817,806	<0,001	Yes
Row 34 vs. Row 59	41,430	8817,436	<0,001	Yes
Row 34 vs. Row 51	41,430	8817,436	<0,001	Yes
Row 34 vs. Row 54	39,880	8816,783	<0,001	Yes
Row 34 vs. Row 47	39,730	8816,720	<0,001	Yes
Row 34 vs. Row 57	36,540	8815,378	<0,001	Yes
Row 34 vs. Row 56	35,150	8814,793	<0,001	Yes
Row 34 vs. Row 42	34,780	8814,637	<0,001	Yes
Row 34 vs. Row 63	31,430	8813,227	<0,001	Yes
Row 34 vs. Row 41	21,160	88 8,905	<0,001	Yes
Row 34 vs. Row 40	17,310	88 7,285	<0,001	Yes
Row 34 vs. Row 65	11,540	88 4,857	0,513	Do Not Test
Row 34 vs. Row 17	11,540	88 4,857	0,513	Do Not Test
Row 34 vs. Row 74	7,700	88 3,241	1,000	Do Not Test
Row 34 vs. Row 48	7,700	88 3,241	1,000	Do Not Test
Row 34 vs. Row 82	3,760	88 1,582	1,000	Do Not Test
Row 34 vs. Row 73	1,930	88 0,812	1,000	Do Not Test
Row 34 vs. Row 81	1,930	88 0,812	1,000	Do Not Test
Row 81 vs. Row 22	95,850	8840,338	<0,001	Yes
Row 81 vs. Row 14	92,650	8838,992	<0,001	Yes
Row 81 vs. Row 30	91,850	8838,655	<0,001	Yes
Row 81 vs. Row 53	89,650	8837,729	<0,001	Yes
Row 81 vs. Row 78	89,250	8837,561	<0,001	Yes
Row 81 vs. Row 87	89,150	8837,519	<0,001	Yes
Row 81 vs. Row 13	89,050	8837,477	<0,001	Yes
Row 81 vs. Row 37	88,650	8837,308	<0,001	Yes
Row 81 vs. Row 5	88,250	8837,140	<0,001	Yes
Row 81 vs. Row 60	85,850	8836,130	<0,001	Yes
Row 81 vs. Row 38	85,150	8835,835	<0,001	Yes
Row 81 vs. Row 29	85,050	8835,793	<0,001	Yes
Row 81 vs. Row 85	84,850	8835,709	<0,001	Yes
Row 81 vs. Row 6	84,350	8835,499	<0,001	Yes

Row 81 vs. Row 52	81,850	8834,446	<0,001	Yes
Row 81 vs. Row 69	81,750	8834,404	<0,001	Yes
Row 81 vs. Row 77	81,350	8834,236	<0,001	Yes
Row 81 vs. Row 61	80,850	8834,026	<0,001	Yes
Row 81 vs. Row 70	80,750	8833,984	<0,001	Yes
Row 81 vs. Row 86	79,650	8833,521	<0,001	Yes
Row 81 vs. Row 44	77,650	8832,679	<0,001	Yes
Row 81 vs. Row 58	76,750	8832,300	<0,001	Yes
Row 81 vs. Row 39	76,150	8832,048	<0,001	Yes
Row 81 vs. Row 31	74,250	8831,248	<0,001	Yes
Row 81 vs. Row 45	73,950	8831,122	<0,001	Yes
Row 81 vs. Row 21	72,950	8830,701	<0,001	Yes
Row 81 vs. Row 15	67,750	8828,512	<0,001	Yes
Row 81 vs. Row 11	66,950	8828,176	<0,001	Yes
Row 81 vs. Row 64	66,550	8828,007	<0,001	Yes
Row 81 vs. Row 10	65,380	8827,515	<0,001	Yes
Row 81 vs. Row 49	65,380	8827,515	<0,001	Yes
Row 81 vs. Row 2	65,380	8827,515	<0,001	Yes
Row 81 vs. Row 12	64,500	8827,145	<0,001	Yes
Row 81 vs. Row 8	62,800	8826,429	<0,001	Yes
Row 81 vs. Row 19	62,550	8826,324	<0,001	Yes
Row 81 vs. Row 62	61,750	8825,987	<0,001	Yes
Row 81 vs. Row 9	61,530	8825,895	<0,001	Yes
Row 81 vs. Row 66	61,530	8825,895	<0,001	Yes
Row 81 vs. Row 23	59,750	8825,146	<0,001	Yes
Row 81 vs. Row 3	59,350	8824,977	<0,001	Yes
Row 81 vs. Row 71	59,350	8824,977	<0,001	Yes
Row 81 vs. Row 50	58,550	8824,641	<0,001	Yes
Row 81 vs. Row 67	57,850	8824,346	<0,001	Yes
Row 81 vs. Row 16	57,800	8824,325	<0,001	Yes
Row 81 vs. Row 24	57,800	8824,325	<0,001	Yes
Row 81 vs. Row 46	56,950	8823,967	<0,001	Yes
Row 81 vs. Row 55	56,150	8823,631	<0,001	Yes
Row 81 vs. Row 36	56,150	8823,631	<0,001	Yes
Row 81 vs. Row 72	56,150	8823,631	<0,001	Yes
Row 81 vs. Row 80	56,150	8823,631	<0,001	Yes
Row 81 vs. Row 79	55,950	8823,546	<0,001	Yes
Row 81 vs. Row 88	54,500	8822,936	<0,001	Yes
Row 81 vs. Row 18	53,840	8822,658	<0,001	Yes
Row 81 vs. Row 43	52,800	8822,221	<0,001	Yes
Row 81 vs. Row 75	51,150	8821,526	<0,001	Yes
Row 81 vs. Row 35	50,550	8821,274	<0,001	Yes
Row 81 vs. Row 1	50,000	8821,042	<0,001	Yes
Row 81 vs. Row 84	49,450	8820,811	<0,001	Yes
Row 81 vs. Row 4	47,800	8820,117	<0,001	Yes
Row 81 vs. Row 20	46,150	8819,422	<0,001	Yes
Row 81 vs. Row 76	44,500	8818,728	<0,001	Yes
Row 81 vs. Row 7	44,350	8818,665	<0,001	Yes
Row 81 vs. Row 83	43,050	8818,118	<0,001	Yes
Row 81 vs. Row 28	42,800	8818,012	<0,001	Yes
Row 81 vs. Row 27	41,650	8817,528	<0,001	Yes
Row 81 vs. Row 68	41,150	8817,318	<0,001	Yes
Row 81 vs. Row 32	40,650	8817,107	<0,001	Yes
Row 81 vs. Row 26	40,380	8816,994	<0,001	Yes
Row 81 vs. Row 59	39,500	8816,624	<0,001	Yes
Row 81 vs. Row 51	39,500	8816,624	<0,001	Yes
Row 81 vs. Row 54	37,950	8815,971	<0,001	Yes
Row 81 vs. Row 47	37,800	8815,908	<0,001	Yes
Row 81 vs. Row 57	34,610	8814,566	<0,001	Yes
Row 81 vs. Row 56	33,220	8813,981	<0,001	Yes
Row 81 vs. Row 42	32,850	8813,825	<0,001	Yes
Row 81 vs. Row 63	29,500	8812,415	<0,001	Yes
Row 81 vs. Row 41	19,230	88 8,093	<0,001	Yes
Row 81 vs. Row 40	15,380	88 6,473	0,013	Yes
Row 81 vs. Row 65	9,610	88 4,044	0,940	Do Not Test
Row 81 vs. Row 17	9,610	88 4,044	0,940	Do Not Test

Row 81 vs. Row 74	5,770	88 2,428	1,000	Do Not Test
Row 81 vs. Row 48	5,770	88 2,428	1,000	Do Not Test
Row 81 vs. Row 82	1,830	88 0,770	1,000	Do Not Test
Row 81 vs. Row 73	0,000	88 0,000	1,000	Do Not Test
Row 73 vs. Row 22	95,850	8840,338	<0,001	Yes
Row 73 vs. Row 14	92,650	8838,992	<0,001	Yes
Row 73 vs. Row 30	91,850	8838,655	<0,001	Yes
Row 73 vs. Row 53	89,650	8837,729	<0,001	Yes
Row 73 vs. Row 78	89,250	8837,561	<0,001	Yes
Row 73 vs. Row 87	89,150	8837,519	<0,001	Yes
Row 73 vs. Row 13	89,050	8837,477	<0,001	Yes
Row 73 vs. Row 37	88,650	8837,308	<0,001	Yes
Row 73 vs. Row 5	88,250	8837,140	<0,001	Yes
Row 73 vs. Row 60	85,850	8836,130	<0,001	Yes
Row 73 vs. Row 38	85,150	8835,835	<0,001	Yes
Row 73 vs. Row 29	85,050	8835,793	<0,001	Yes
Row 73 vs. Row 85	84,850	8835,709	<0,001	Yes
Row 73 vs. Row 6	84,350	8835,499	<0,001	Yes
Row 73 vs. Row 52	81,850	8834,446	<0,001	Yes
Row 73 vs. Row 69	81,750	8834,404	<0,001	Yes
Row 73 vs. Row 77	81,350	8834,236	<0,001	Yes
Row 73 vs. Row 61	80,850	8834,026	<0,001	Yes
Row 73 vs. Row 70	80,750	8833,984	<0,001	Yes
Row 73 vs. Row 86	79,650	8833,521	<0,001	Yes
Row 73 vs. Row 44	77,650	8832,679	<0,001	Yes
Row 73 vs. Row 58	76,750	8832,300	<0,001	Yes
Row 73 vs. Row 39	76,150	8832,048	<0,001	Yes
Row 73 vs. Row 31	74,250	8831,248	<0,001	Yes
Row 73 vs. Row 45	73,950	8831,122	<0,001	Yes
Row 73 vs. Row 21	72,950	8830,701	<0,001	Yes
Row 73 vs. Row 15	67,750	8828,512	<0,001	Yes
Row 73 vs. Row 11	66,950	8828,176	<0,001	Yes
Row 73 vs. Row 64	66,550	8828,007	<0,001	Yes
Row 73 vs. Row 10	65,380	8827,515	<0,001	Yes
Row 73 vs. Row 49	65,380	8827,515	<0,001	Yes
Row 73 vs. Row 2	65,380	8827,515	<0,001	Yes
Row 73 vs. Row 12	64,500	8827,145	<0,001	Yes
Row 73 vs. Row 8	62,800	8826,429	<0,001	Yes
Row 73 vs. Row 19	62,550	8826,324	<0,001	Yes
Row 73 vs. Row 62	61,750	8825,987	<0,001	Yes
Row 73 vs. Row 9	61,530	8825,895	<0,001	Yes
Row 73 vs. Row 66	61,530	8825,895	<0,001	Yes
Row 73 vs. Row 23	59,750	8825,146	<0,001	Yes
Row 73 vs. Row 3	59,350	8824,977	<0,001	Yes
Row 73 vs. Row 71	59,350	8824,977	<0,001	Yes
Row 73 vs. Row 50	58,550	8824,641	<0,001	Yes
Row 73 vs. Row 67	57,850	8824,346	<0,001	Yes
Row 73 vs. Row 16	57,800	8824,325	<0,001	Yes
Row 73 vs. Row 24	57,800	8824,325	<0,001	Yes
Row 73 vs. Row 46	56,950	8823,967	<0,001	Yes
Row 73 vs. Row 55	56,150	8823,631	<0,001	Yes
Row 73 vs. Row 36	56,150	8823,631	<0,001	Yes
Row 73 vs. Row 72	56,150	8823,631	<0,001	Yes
Row 73 vs. Row 80	56,150	8823,631	<0,001	Yes
Row 73 vs. Row 79	55,950	8823,546	<0,001	Yes
Row 73 vs. Row 88	54,500	8822,936	<0,001	Yes
Row 73 vs. Row 18	53,840	8822,658	<0,001	Yes
Row 73 vs. Row 43	52,800	8822,221	<0,001	Yes
Row 73 vs. Row 75	51,150	8821,526	<0,001	Yes
Row 73 vs. Row 35	50,550	8821,274	<0,001	Yes
Row 73 vs. Row 1	50,000	8821,042	<0,001	Yes
Row 73 vs. Row 84	49,450	8820,811	<0,001	Yes
Row 73 vs. Row 4	47,800	8820,117	<0,001	Yes
Row 73 vs. Row 20	46,150	8819,422	<0,001	Yes
Row 73 vs. Row 76	44,500	8818,728	<0,001	Yes
Row 73 vs. Row 7	44,350	8818,665	<0,001	Yes

Row 73 vs. Row 83	43,050	8818,118	<0,001	Yes
Row 73 vs. Row 28	42,800	8818,012	<0,001	Yes
Row 73 vs. Row 27	41,650	8817,528	<0,001	Yes
Row 73 vs. Row 68	41,150	8817,318	<0,001	Yes
Row 73 vs. Row 32	40,650	8817,107	<0,001	Yes
Row 73 vs. Row 26	40,380	8816,994	<0,001	Yes
Row 73 vs. Row 59	39,500	8816,624	<0,001	Yes
Row 73 vs. Row 51	39,500	8816,624	<0,001	Yes
Row 73 vs. Row 54	37,950	8815,971	<0,001	Yes
Row 73 vs. Row 47	37,800	8815,908	<0,001	Yes
Row 73 vs. Row 57	34,610	8814,566	<0,001	Yes
Row 73 vs. Row 56	33,220	8813,981	<0,001	Yes
Row 73 vs. Row 42	32,850	8813,825	<0,001	Yes
Row 73 vs. Row 63	29,500	8812,415	<0,001	Yes
Row 73 vs. Row 41	19,230	88 8,093	<0,001	Yes
Row 73 vs. Row 40	15,380	88 6,473	0,013	Yes
Row 73 vs. Row 65	9,610	88 4,044	0,940	Do Not Test
Row 73 vs. Row 17	9,610	88 4,044	0,940	Do Not Test
Row 73 vs. Row 74	5,770	88 2,428	1,000	Do Not Test
Row 73 vs. Row 48	5,770	88 2,428	1,000	Do Not Test
Row 73 vs. Row 82	1,830	88 0,770	1,000	Do Not Test
Row 82 vs. Row 22	94,020	8839,568	<0,001	Yes
Row 82 vs. Row 14	90,820	8838,221	<0,001	Yes
Row 82 vs. Row 30	90,020	8837,885	<0,001	Yes
Row 82 vs. Row 53	87,820	8836,959	<0,001	Yes
Row 82 vs. Row 78	87,420	8836,791	<0,001	Yes
Row 82 vs. Row 87	87,320	8836,748	<0,001	Yes
Row 82 vs. Row 13	87,220	8836,706	<0,001	Yes
Row 82 vs. Row 37	86,820	8836,538	<0,001	Yes
Row 82 vs. Row 5	86,420	8836,370	<0,001	Yes
Row 82 vs. Row 60	84,020	8835,360	<0,001	Yes
Row 82 vs. Row 38	83,320	8835,065	<0,001	Yes
Row 82 vs. Row 29	83,220	8835,023	<0,001	Yes
Row 82 vs. Row 85	83,020	8834,939	<0,001	Yes
Row 82 vs. Row 6	82,520	8834,728	<0,001	Yes
Row 82 vs. Row 52	80,020	8833,676	<0,001	Yes
Row 82 vs. Row 69	79,920	8833,634	<0,001	Yes
Row 82 vs. Row 77	79,520	8833,466	<0,001	Yes
Row 82 vs. Row 61	79,020	8833,255	<0,001	Yes
Row 82 vs. Row 70	78,920	8833,213	<0,001	Yes
Row 82 vs. Row 86	77,820	8832,750	<0,001	Yes
Row 82 vs. Row 44	75,820	8831,909	<0,001	Yes
Row 82 vs. Row 58	74,920	8831,530	<0,001	Yes
Row 82 vs. Row 39	74,320	8831,277	<0,001	Yes
Row 82 vs. Row 31	72,420	8830,478	<0,001	Yes
Row 82 vs. Row 45	72,120	8830,352	<0,001	Yes
Row 82 vs. Row 21	71,120	8829,931	<0,001	Yes
Row 82 vs. Row 15	65,920	8827,742	<0,001	Yes
Row 82 vs. Row 11	65,120	8827,406	<0,001	Yes
Row 82 vs. Row 64	64,720	8827,237	<0,001	Yes
Row 82 vs. Row 10	63,550	8826,745	<0,001	Yes
Row 82 vs. Row 49	63,550	8826,745	<0,001	Yes
Row 82 vs. Row 2	63,550	8826,745	<0,001	Yes
Row 82 vs. Row 12	62,670	8826,375	<0,001	Yes
Row 82 vs. Row 8	60,970	8825,659	<0,001	Yes
Row 82 vs. Row 19	60,720	8825,554	<0,001	Yes
Row 82 vs. Row 62	59,920	8825,217	<0,001	Yes
Row 82 vs. Row 9	59,700	8825,125	<0,001	Yes
Row 82 vs. Row 66	59,700	8825,125	<0,001	Yes
Row 82 vs. Row 23	57,920	8824,376	<0,001	Yes
Row 82 vs. Row 3	57,520	8824,207	<0,001	Yes
Row 82 vs. Row 71	57,520	8824,207	<0,001	Yes
Row 82 vs. Row 50	56,720	8823,871	<0,001	Yes
Row 82 vs. Row 67	56,020	8823,576	<0,001	Yes
Row 82 vs. Row 16	55,970	8823,555	<0,001	Yes
Row 82 vs. Row 24	55,970	8823,555	<0,001	Yes

Row 82 vs. Row 46	55,120	8823,197	<0,001	Yes
Row 82 vs. Row 55	54,320	8822,860	<0,001	Yes
Row 82 vs. Row 36	54,320	8822,860	<0,001	Yes
Row 82 vs. Row 72	54,320	8822,860	<0,001	Yes
Row 82 vs. Row 80	54,320	8822,860	<0,001	Yes
Row 82 vs. Row 79	54,120	8822,776	<0,001	Yes
Row 82 vs. Row 88	52,670	8822,166	<0,001	Yes
Row 82 vs. Row 18	52,010	8821,888	<0,001	Yes
Row 82 vs. Row 43	50,970	8821,451	<0,001	Yes
Row 82 vs. Row 75	49,320	8820,756	<0,001	Yes
Row 82 vs. Row 35	48,720	8820,504	<0,001	Yes
Row 82 vs. Row 1	48,170	8820,272	<0,001	Yes
Row 82 vs. Row 84	47,620	8820,041	<0,001	Yes
Row 82 vs. Row 4	45,970	8819,346	<0,001	Yes
Row 82 vs. Row 20	44,320	8818,652	<0,001	Yes
Row 82 vs. Row 76	42,670	8817,958	<0,001	Yes
Row 82 vs. Row 7	42,520	8817,894	<0,001	Yes
Row 82 vs. Row 83	41,220	8817,347	<0,001	Yes
Row 82 vs. Row 28	40,970	8817,242	<0,001	Yes
Row 82 vs. Row 27	39,820	8816,758	<0,001	Yes
Row 82 vs. Row 68	39,320	8816,548	<0,001	Yes
Row 82 vs. Row 32	38,820	8816,337	<0,001	Yes
Row 82 vs. Row 26	38,550	8816,224	<0,001	Yes
Row 82 vs. Row 59	37,670	8815,853	<0,001	Yes
Row 82 vs. Row 51	37,670	8815,853	<0,001	Yes
Row 82 vs. Row 54	36,120	8815,201	<0,001	Yes
Row 82 vs. Row 47	35,970	8815,138	<0,001	Yes
Row 82 vs. Row 57	32,780	8813,795	<0,001	Yes
Row 82 vs. Row 56	31,390	8813,210	<0,001	Yes
Row 82 vs. Row 42	31,020	8813,055	<0,001	Yes
Row 82 vs. Row 63	27,670	8811,645	<0,001	Yes
Row 82 vs. Row 41	17,400	88 7,323	<0,001	Yes
Row 82 vs. Row 40	13,550	88 5,702	0,107	No
Row 82 vs. Row 65	7,780	88 3,274	1,000	Do Not Test
Row 82 vs. Row 17	7,780	88 3,274	1,000	Do Not Test
Row 82 vs. Row 74	3,940	88 1,658	1,000	Do Not Test
Row 82 vs. Row 48	3,940	88 1,658	1,000	Do Not Test
Row 48 vs. Row 22	90,080	8837,910	<0,001	Yes
Row 48 vs. Row 14	86,880	8836,563	<0,001	Yes
Row 48 vs. Row 30	86,080	8836,227	<0,001	Yes
Row 48 vs. Row 53	83,880	8835,301	<0,001	Yes
Row 48 vs. Row 78	83,480	8835,132	<0,001	Yes
Row 48 vs. Row 87	83,380	8835,090	<0,001	Yes
Row 48 vs. Row 13	83,280	8835,048	<0,001	Yes
Row 48 vs. Row 37	82,880	8834,880	<0,001	Yes
Row 48 vs. Row 5	82,480	8834,712	<0,001	Yes
Row 48 vs. Row 60	80,080	8833,702	<0,001	Yes
Row 48 vs. Row 38	79,380	8833,407	<0,001	Yes
Row 48 vs. Row 29	79,280	8833,365	<0,001	Yes
Row 48 vs. Row 85	79,080	8833,281	<0,001	Yes
Row 48 vs. Row 6	78,580	8833,070	<0,001	Yes
Row 48 vs. Row 52	76,080	8832,018	<0,001	Yes
Row 48 vs. Row 69	75,980	8831,976	<0,001	Yes
Row 48 vs. Row 77	75,580	8831,808	<0,001	Yes
Row 48 vs. Row 61	75,080	8831,597	<0,001	Yes
Row 48 vs. Row 70	74,980	8831,555	<0,001	Yes
Row 48 vs. Row 86	73,880	8831,092	<0,001	Yes
Row 48 vs. Row 44	71,880	8830,251	<0,001	Yes
Row 48 vs. Row 58	70,980	8829,872	<0,001	Yes
Row 48 vs. Row 39	70,380	8829,619	<0,001	Yes
Row 48 vs. Row 31	68,480	8828,820	<0,001	Yes
Row 48 vs. Row 45	68,180	8828,693	<0,001	Yes
Row 48 vs. Row 21	67,180	8828,273	<0,001	Yes
Row 48 vs. Row 15	61,980	8826,084	<0,001	Yes
Row 48 vs. Row 11	61,180	8825,748	<0,001	Yes
Row 48 vs. Row 64	60,780	8825,579	<0,001	Yes

Row 48 vs. Row 10	59,610	8825,087	<0,001	Yes
Row 48 vs. Row 49	59,610	8825,087	<0,001	Yes
Row 48 vs. Row 2	59,610	8825,087	<0,001	Yes
Row 48 vs. Row 12	58,730	8824,716	<0,001	Yes
Row 48 vs. Row 8	57,030	8824,001	<0,001	Yes
Row 48 vs. Row 19	56,780	8823,896	<0,001	Yes
Row 48 vs. Row 62	55,980	8823,559	<0,001	Yes
Row 48 vs. Row 9	55,760	8823,467	<0,001	Yes
Row 48 vs. Row 66	55,760	8823,467	<0,001	Yes
Row 48 vs. Row 23	53,980	8822,717	<0,001	Yes
Row 48 vs. Row 3	53,580	8822,549	<0,001	Yes
Row 48 vs. Row 71	53,580	8822,549	<0,001	Yes
Row 48 vs. Row 50	52,780	8822,212	<0,001	Yes
Row 48 vs. Row 67	52,080	8821,918	<0,001	Yes
Row 48 vs. Row 16	52,030	8821,897	<0,001	Yes
Row 48 vs. Row 24	52,030	8821,897	<0,001	Yes
Row 48 vs. Row 46	51,180	8821,539	<0,001	Yes
Row 48 vs. Row 55	50,380	8821,202	<0,001	Yes
Row 48 vs. Row 36	50,380	8821,202	<0,001	Yes
Row 48 vs. Row 72	50,380	8821,202	<0,001	Yes
Row 48 vs. Row 80	50,380	8821,202	<0,001	Yes
Row 48 vs. Row 79	50,180	8821,118	<0,001	Yes
Row 48 vs. Row 88	48,730	8820,508	<0,001	Yes
Row 48 vs. Row 18	48,070	8820,230	<0,001	Yes
Row 48 vs. Row 43	47,030	8819,793	<0,001	Yes
Row 48 vs. Row 75	45,380	8819,098	<0,001	Yes
Row 48 vs. Row 35	44,780	8818,846	<0,001	Yes
Row 48 vs. Row 1	44,230	8818,614	<0,001	Yes
Row 48 vs. Row 84	43,680	8818,383	<0,001	Yes
Row 48 vs. Row 4	42,030	8817,688	<0,001	Yes
Row 48 vs. Row 20	40,380	8816,994	<0,001	Yes
Row 48 vs. Row 76	38,730	8816,299	<0,001	Yes
Row 48 vs. Row 7	38,580	8816,236	<0,001	Yes
Row 48 vs. Row 83	37,280	8815,689	<0,001	Yes
Row 48 vs. Row 28	37,030	8815,584	<0,001	Yes
Row 48 vs. Row 27	35,880	8815,100	<0,001	Yes
Row 48 vs. Row 68	35,380	8814,890	<0,001	Yes
Row 48 vs. Row 32	34,880	8814,679	<0,001	Yes
Row 48 vs. Row 26	34,610	8814,566	<0,001	Yes
Row 48 vs. Row 59	33,730	8814,195	<0,001	Yes
Row 48 vs. Row 51	33,730	8814,195	<0,001	Yes
Row 48 vs. Row 54	32,180	8813,543	<0,001	Yes
Row 48 vs. Row 47	32,030	8813,480	<0,001	Yes
Row 48 vs. Row 57	28,840	8812,137	<0,001	Yes
Row 48 vs. Row 56	27,450	8811,552	<0,001	Yes
Row 48 vs. Row 42	27,080	8811,397	<0,001	Yes
Row 48 vs. Row 63	23,730	88 9,987	<0,001	Yes
Row 48 vs. Row 41	13,460	88 5,665	0,117	No
Row 48 vs. Row 40	9,610	88 4,044	0,940	Do Not Test
Row 48 vs. Row 65	3,840	88 1,616	1,000	Do Not Test
Row 48 vs. Row 17	3,840	88 1,616	1,000	Do Not Test
Row 48 vs. Row 74	0,000	88 0,000	1,000	Do Not Test
Row 74 vs. Row 22	90,080	8837,910	<0,001	Yes
Row 74 vs. Row 14	86,880	8836,563	<0,001	Yes
Row 74 vs. Row 30	86,080	8836,227	<0,001	Yes
Row 74 vs. Row 53	83,880	8835,301	<0,001	Yes
Row 74 vs. Row 78	83,480	8835,132	<0,001	Yes
Row 74 vs. Row 87	83,380	8835,090	<0,001	Yes
Row 74 vs. Row 13	83,280	8835,048	<0,001	Yes
Row 74 vs. Row 37	82,880	8834,880	<0,001	Yes
Row 74 vs. Row 5	82,480	8834,712	<0,001	Yes
Row 74 vs. Row 60	80,080	8833,702	<0,001	Yes
Row 74 vs. Row 38	79,380	8833,407	<0,001	Yes
Row 74 vs. Row 29	79,280	8833,365	<0,001	Yes
Row 74 vs. Row 85	79,080	8833,281	<0,001	Yes
Row 74 vs. Row 6	78,580	8833,070	<0,001	Yes

Row 74 vs. Row 52	76,080	8832,018	<0,001	Yes
Row 74 vs. Row 69	75,980	8831,976	<0,001	Yes
Row 74 vs. Row 77	75,580	8831,808	<0,001	Yes
Row 74 vs. Row 61	75,080	8831,597	<0,001	Yes
Row 74 vs. Row 70	74,980	8831,555	<0,001	Yes
Row 74 vs. Row 86	73,880	8831,092	<0,001	Yes
Row 74 vs. Row 44	71,880	8830,251	<0,001	Yes
Row 74 vs. Row 58	70,980	8829,872	<0,001	Yes
Row 74 vs. Row 39	70,380	8829,619	<0,001	Yes
Row 74 vs. Row 31	68,480	8828,820	<0,001	Yes
Row 74 vs. Row 45	68,180	8828,693	<0,001	Yes
Row 74 vs. Row 21	67,180	8828,273	<0,001	Yes
Row 74 vs. Row 15	61,980	8826,084	<0,001	Yes
Row 74 vs. Row 11	61,180	8825,748	<0,001	Yes
Row 74 vs. Row 64	60,780	8825,579	<0,001	Yes
Row 74 vs. Row 10	59,610	8825,087	<0,001	Yes
Row 74 vs. Row 49	59,610	8825,087	<0,001	Yes
Row 74 vs. Row 2	59,610	8825,087	<0,001	Yes
Row 74 vs. Row 12	58,730	8824,716	<0,001	Yes
Row 74 vs. Row 8	57,030	8824,001	<0,001	Yes
Row 74 vs. Row 19	56,780	8823,896	<0,001	Yes
Row 74 vs. Row 62	55,980	8823,559	<0,001	Yes
Row 74 vs. Row 9	55,760	8823,467	<0,001	Yes
Row 74 vs. Row 66	55,760	8823,467	<0,001	Yes
Row 74 vs. Row 23	53,980	8822,717	<0,001	Yes
Row 74 vs. Row 3	53,580	8822,549	<0,001	Yes
Row 74 vs. Row 71	53,580	8822,549	<0,001	Yes
Row 74 vs. Row 50	52,780	8822,212	<0,001	Yes
Row 74 vs. Row 67	52,080	8821,918	<0,001	Yes
Row 74 vs. Row 16	52,030	8821,897	<0,001	Yes
Row 74 vs. Row 24	52,030	8821,897	<0,001	Yes
Row 74 vs. Row 46	51,180	8821,539	<0,001	Yes
Row 74 vs. Row 55	50,380	8821,202	<0,001	Yes
Row 74 vs. Row 36	50,380	8821,202	<0,001	Yes
Row 74 vs. Row 72	50,380	8821,202	<0,001	Yes
Row 74 vs. Row 80	50,380	8821,202	<0,001	Yes
Row 74 vs. Row 79	50,180	8821,118	<0,001	Yes
Row 74 vs. Row 88	48,730	8820,508	<0,001	Yes
Row 74 vs. Row 18	48,070	8820,230	<0,001	Yes
Row 74 vs. Row 43	47,030	8819,793	<0,001	Yes
Row 74 vs. Row 75	45,380	8819,098	<0,001	Yes
Row 74 vs. Row 35	44,780	8818,846	<0,001	Yes
Row 74 vs. Row 1	44,230	8818,614	<0,001	Yes
Row 74 vs. Row 84	43,680	8818,383	<0,001	Yes
Row 74 vs. Row 4	42,030	8817,688	<0,001	Yes
Row 74 vs. Row 20	40,380	8816,994	<0,001	Yes
Row 74 vs. Row 76	38,730	8816,299	<0,001	Yes
Row 74 vs. Row 7	38,580	8816,236	<0,001	Yes
Row 74 vs. Row 83	37,280	8815,689	<0,001	Yes
Row 74 vs. Row 28	37,030	8815,584	<0,001	Yes
Row 74 vs. Row 27	35,880	8815,100	<0,001	Yes
Row 74 vs. Row 68	35,380	8814,890	<0,001	Yes
Row 74 vs. Row 32	34,880	8814,679	<0,001	Yes
Row 74 vs. Row 26	34,610	8814,566	<0,001	Yes
Row 74 vs. Row 59	33,730	8814,195	<0,001	Yes
Row 74 vs. Row 51	33,730	8814,195	<0,001	Yes
Row 74 vs. Row 54	32,180	8813,543	<0,001	Yes
Row 74 vs. Row 47	32,030	8813,480	<0,001	Yes
Row 74 vs. Row 57	28,840	8812,137	<0,001	Yes
Row 74 vs. Row 56	27,450	8811,552	<0,001	Yes
Row 74 vs. Row 42	27,080	8811,397	<0,001	Yes
Row 74 vs. Row 63	23,730	88 9,987	<0,001	Yes
Row 74 vs. Row 41	13,460	88 5,665	0,117	Do Not Test
Row 74 vs. Row 40	9,610	88 4,044	0,940	Do Not Test
Row 74 vs. Row 65	3,840	88 1,616	1,000	Do Not Test
Row 74 vs. Row 17	3,840	88 1,616	1,000	Do Not Test

Row 17 vs. Row 22	86,240	8836,294	<0,001	Yes
Row 17 vs. Row 14	83,040	8834,947	<0,001	Yes
Row 17 vs. Row 30	82,240	8834,611	<0,001	Yes
Row 17 vs. Row 53	80,040	8833,685	<0,001	Yes
Row 17 vs. Row 78	79,640	8833,516	<0,001	Yes
Row 17 vs. Row 87	79,540	8833,474	<0,001	Yes
Row 17 vs. Row 13	79,440	8833,432	<0,001	Yes
Row 17 vs. Row 37	79,040	8833,264	<0,001	Yes
Row 17 vs. Row 5	78,640	8833,096	<0,001	Yes
Row 17 vs. Row 60	76,240	8832,085	<0,001	Yes
Row 17 vs. Row 38	75,540	8831,791	<0,001	Yes
Row 17 vs. Row 29	75,440	8831,749	<0,001	Yes
Row 17 vs. Row 85	75,240	8831,665	<0,001	Yes
Row 17 vs. Row 6	74,740	8831,454	<0,001	Yes
Row 17 vs. Row 52	72,240	8830,402	<0,001	Yes
Row 17 vs. Row 69	72,140	8830,360	<0,001	Yes
Row 17 vs. Row 77	71,740	8830,192	<0,001	Yes
Row 17 vs. Row 61	71,240	8829,981	<0,001	Yes
Row 17 vs. Row 70	71,140	8829,939	<0,001	Yes
Row 17 vs. Row 86	70,040	8829,476	<0,001	Yes
Row 17 vs. Row 44	68,040	8828,635	<0,001	Yes
Row 17 vs. Row 58	67,140	8828,256	<0,001	Yes
Row 17 vs. Row 39	66,540	8828,003	<0,001	Yes
Row 17 vs. Row 31	64,640	8827,204	<0,001	Yes
Row 17 vs. Row 45	64,340	8827,077	<0,001	Yes
Row 17 vs. Row 21	63,340	8826,657	<0,001	Yes
Row 17 vs. Row 15	58,140	8824,468	<0,001	Yes
Row 17 vs. Row 11	57,340	8824,131	<0,001	Yes
Row 17 vs. Row 64	56,940	8823,963	<0,001	Yes
Row 17 vs. Row 10	55,770	8823,471	<0,001	Yes
Row 17 vs. Row 49	55,770	8823,471	<0,001	Yes
Row 17 vs. Row 2	55,770	8823,471	<0,001	Yes
Row 17 vs. Row 12	54,890	8823,100	<0,001	Yes
Row 17 vs. Row 8	53,190	8822,385	<0,001	Yes
Row 17 vs. Row 19	52,940	8822,280	<0,001	Yes
Row 17 vs. Row 62	52,140	8821,943	<0,001	Yes
Row 17 vs. Row 9	51,920	8821,850	<0,001	Yes
Row 17 vs. Row 66	51,920	8821,850	<0,001	Yes
Row 17 vs. Row 23	50,140	8821,101	<0,001	Yes
Row 17 vs. Row 3	49,740	8820,933	<0,001	Yes
Row 17 vs. Row 71	49,740	8820,933	<0,001	Yes
Row 17 vs. Row 50	48,940	8820,596	<0,001	Yes
Row 17 vs. Row 67	48,240	8820,302	<0,001	Yes
Row 17 vs. Row 16	48,190	8820,281	<0,001	Yes
Row 17 vs. Row 24	48,190	8820,281	<0,001	Yes
Row 17 vs. Row 46	47,340	8819,923	<0,001	Yes
Row 17 vs. Row 55	46,540	8819,586	<0,001	Yes
Row 17 vs. Row 36	46,540	8819,586	<0,001	Yes
Row 17 vs. Row 72	46,540	8819,586	<0,001	Yes
Row 17 vs. Row 80	46,540	8819,586	<0,001	Yes
Row 17 vs. Row 79	46,340	8819,502	<0,001	Yes
Row 17 vs. Row 88	44,890	8818,892	<0,001	Yes
Row 17 vs. Row 18	44,230	8818,614	<0,001	Yes
Row 17 vs. Row 43	43,190	8818,176	<0,001	Yes
Row 17 vs. Row 75	41,540	8817,482	<0,001	Yes
Row 17 vs. Row 35	40,940	8817,230	<0,001	Yes
Row 17 vs. Row 1	40,390	8816,998	<0,001	Yes
Row 17 vs. Row 84	39,840	8816,767	<0,001	Yes
Row 17 vs. Row 4	38,190	8816,072	<0,001	Yes
Row 17 vs. Row 20	36,540	8815,378	<0,001	Yes
Row 17 vs. Row 76	34,890	8814,683	<0,001	Yes
Row 17 vs. Row 7	34,740	8814,620	<0,001	Yes
Row 17 vs. Row 83	33,440	8814,073	<0,001	Yes
Row 17 vs. Row 28	33,190	8813,968	<0,001	Yes
Row 17 vs. Row 27	32,040	8813,484	<0,001	Yes
Row 17 vs. Row 68	31,540	8813,274	<0,001	Yes

Row 17 vs. Row 32	31,040	8813,063	<0,001	Yes
Row 17 vs. Row 26	30,770	8812,950	<0,001	Yes
Row 17 vs. Row 59	29,890	8812,579	<0,001	Yes
Row 17 vs. Row 51	29,890	8812,579	<0,001	Yes
Row 17 vs. Row 54	28,340	8811,927	<0,001	Yes
Row 17 vs. Row 47	28,190	8811,864	<0,001	Yes
Row 17 vs. Row 57	25,000	8810,521	<0,001	Yes
Row 17 vs. Row 56	23,610	88 9,936	<0,001	Yes
Row 17 vs. Row 42	23,240	88 9,781	<0,001	Yes
Row 17 vs. Row 63	19,890	88 8,371	<0,001	Yes
Row 17 vs. Row 41	9,620	88 4,049	0,939	Do Not Test
Row 17 vs. Row 40	5,770	88 2,428	1,000	Do Not Test
Row 17 vs. Row 65	0,000	88 0,000	1,000	Do Not Test
Row 65 vs. Row 22	86,240	8836,294	<0,001	Yes
Row 65 vs. Row 14	83,040	8834,947	<0,001	Yes
Row 65 vs. Row 30	82,240	8834,611	<0,001	Yes
Row 65 vs. Row 53	80,040	8833,685	<0,001	Yes
Row 65 vs. Row 78	79,640	8833,516	<0,001	Yes
Row 65 vs. Row 87	79,540	8833,474	<0,001	Yes
Row 65 vs. Row 13	79,440	8833,432	<0,001	Yes
Row 65 vs. Row 37	79,040	8833,264	<0,001	Yes
Row 65 vs. Row 5	78,640	8833,096	<0,001	Yes
Row 65 vs. Row 60	76,240	8832,085	<0,001	Yes
Row 65 vs. Row 38	75,540	8831,791	<0,001	Yes
Row 65 vs. Row 29	75,440	8831,749	<0,001	Yes
Row 65 vs. Row 85	75,240	8831,665	<0,001	Yes
Row 65 vs. Row 6	74,740	8831,454	<0,001	Yes
Row 65 vs. Row 52	72,240	8830,402	<0,001	Yes
Row 65 vs. Row 69	72,140	8830,360	<0,001	Yes
Row 65 vs. Row 77	71,740	8830,192	<0,001	Yes
Row 65 vs. Row 61	71,240	8829,981	<0,001	Yes
Row 65 vs. Row 70	71,140	8829,939	<0,001	Yes
Row 65 vs. Row 86	70,040	8829,476	<0,001	Yes
Row 65 vs. Row 44	68,040	8828,635	<0,001	Yes
Row 65 vs. Row 58	67,140	8828,256	<0,001	Yes
Row 65 vs. Row 39	66,540	8828,003	<0,001	Yes
Row 65 vs. Row 31	64,640	8827,204	<0,001	Yes
Row 65 vs. Row 45	64,340	8827,077	<0,001	Yes
Row 65 vs. Row 21	63,340	8826,657	<0,001	Yes
Row 65 vs. Row 15	58,140	8824,468	<0,001	Yes
Row 65 vs. Row 11	57,340	8824,131	<0,001	Yes
Row 65 vs. Row 64	56,940	8823,963	<0,001	Yes
Row 65 vs. Row 10	55,770	8823,471	<0,001	Yes
Row 65 vs. Row 49	55,770	8823,471	<0,001	Yes
Row 65 vs. Row 2	55,770	8823,471	<0,001	Yes
Row 65 vs. Row 12	54,890	8823,100	<0,001	Yes
Row 65 vs. Row 8	53,190	8822,385	<0,001	Yes
Row 65 vs. Row 19	52,940	8822,280	<0,001	Yes
Row 65 vs. Row 62	52,140	8821,943	<0,001	Yes
Row 65 vs. Row 9	51,920	8821,850	<0,001	Yes
Row 65 vs. Row 66	51,920	8821,850	<0,001	Yes
Row 65 vs. Row 23	50,140	8821,101	<0,001	Yes
Row 65 vs. Row 3	49,740	8820,933	<0,001	Yes
Row 65 vs. Row 71	49,740	8820,933	<0,001	Yes
Row 65 vs. Row 50	48,940	8820,596	<0,001	Yes
Row 65 vs. Row 67	48,240	8820,302	<0,001	Yes
Row 65 vs. Row 16	48,190	8820,281	<0,001	Yes
Row 65 vs. Row 24	48,190	8820,281	<0,001	Yes
Row 65 vs. Row 46	47,340	8819,923	<0,001	Yes
Row 65 vs. Row 55	46,540	8819,586	<0,001	Yes
Row 65 vs. Row 36	46,540	8819,586	<0,001	Yes
Row 65 vs. Row 72	46,540	8819,586	<0,001	Yes
Row 65 vs. Row 80	46,540	8819,586	<0,001	Yes
Row 65 vs. Row 79	46,340	8819,502	<0,001	Yes
Row 65 vs. Row 88	44,890	8818,892	<0,001	Yes
Row 65 vs. Row 18	44,230	8818,614	<0,001	Yes

Row 65 vs. Row 43	43,190	8818,176	<0,001	Yes
Row 65 vs. Row 75	41,540	8817,482	<0,001	Yes
Row 65 vs. Row 35	40,940	8817,230	<0,001	Yes
Row 65 vs. Row 1	40,390	8816,998	<0,001	Yes
Row 65 vs. Row 84	39,840	8816,767	<0,001	Yes
Row 65 vs. Row 4	38,190	8816,072	<0,001	Yes
Row 65 vs. Row 20	36,540	8815,378	<0,001	Yes
Row 65 vs. Row 76	34,890	8814,683	<0,001	Yes
Row 65 vs. Row 7	34,740	8814,620	<0,001	Yes
Row 65 vs. Row 83	33,440	8814,073	<0,001	Yes
Row 65 vs. Row 28	33,190	8813,968	<0,001	Yes
Row 65 vs. Row 27	32,040	8813,484	<0,001	Yes
Row 65 vs. Row 68	31,540	8813,274	<0,001	Yes
Row 65 vs. Row 32	31,040	8813,063	<0,001	Yes
Row 65 vs. Row 26	30,770	8812,950	<0,001	Yes
Row 65 vs. Row 59	29,890	8812,579	<0,001	Yes
Row 65 vs. Row 51	29,890	8812,579	<0,001	Yes
Row 65 vs. Row 54	28,340	8811,927	<0,001	Yes
Row 65 vs. Row 47	28,190	8811,864	<0,001	Yes
Row 65 vs. Row 57	25,000	8810,521	<0,001	Yes
Row 65 vs. Row 56	23,610	88 9,936	<0,001	Yes
Row 65 vs. Row 42	23,240	88 9,781	<0,001	Yes
Row 65 vs. Row 63	19,890	88 8,371	<0,001	Yes
Row 65 vs. Row 41	9,620	88 4,049	0,939	Do Not Test
Row 65 vs. Row 40	5,770	88 2,428	1,000	Do Not Test
Row 40 vs. Row 22	80,470	8833,866	<0,001	Yes
Row 40 vs. Row 14	77,270	8832,519	<0,001	Yes
Row 40 vs. Row 30	76,470	8832,182	<0,001	Yes
Row 40 vs. Row 53	74,270	8831,256	<0,001	Yes
Row 40 vs. Row 78	73,870	8831,088	<0,001	Yes
Row 40 vs. Row 87	73,770	8831,046	<0,001	Yes
Row 40 vs. Row 13	73,670	8831,004	<0,001	Yes
Row 40 vs. Row 37	73,270	8830,836	<0,001	Yes
Row 40 vs. Row 5	72,870	8830,667	<0,001	Yes
Row 40 vs. Row 60	70,470	8829,657	<0,001	Yes
Row 40 vs. Row 38	69,770	8829,363	<0,001	Yes
Row 40 vs. Row 29	69,670	8829,321	<0,001	Yes
Row 40 vs. Row 85	69,470	8829,236	<0,001	Yes
Row 40 vs. Row 6	68,970	8829,026	<0,001	Yes
Row 40 vs. Row 52	66,470	8827,974	<0,001	Yes
Row 40 vs. Row 69	66,370	8827,932	<0,001	Yes
Row 40 vs. Row 77	65,970	8827,763	<0,001	Yes
Row 40 vs. Row 61	65,470	8827,553	<0,001	Yes
Row 40 vs. Row 70	65,370	8827,511	<0,001	Yes
Row 40 vs. Row 86	64,270	8827,048	<0,001	Yes
Row 40 vs. Row 44	62,270	8826,206	<0,001	Yes
Row 40 vs. Row 58	61,370	8825,827	<0,001	Yes
Row 40 vs. Row 39	60,770	8825,575	<0,001	Yes
Row 40 vs. Row 31	58,870	8824,775	<0,001	Yes
Row 40 vs. Row 45	58,570	8824,649	<0,001	Yes
Row 40 vs. Row 21	57,570	8824,228	<0,001	Yes
Row 40 vs. Row 15	52,370	8822,040	<0,001	Yes
Row 40 vs. Row 11	51,570	8821,703	<0,001	Yes
Row 40 vs. Row 64	51,170	8821,535	<0,001	Yes
Row 40 vs. Row 10	50,000	8821,042	<0,001	Yes
Row 40 vs. Row 49	50,000	8821,042	<0,001	Yes
Row 40 vs. Row 2	50,000	8821,042	<0,001	Yes
Row 40 vs. Row 12	49,120	8820,672	<0,001	Yes
Row 40 vs. Row 8	47,420	8819,957	<0,001	Yes
Row 40 vs. Row 19	47,170	8819,851	<0,001	Yes
Row 40 vs. Row 62	46,370	8819,515	<0,001	Yes
Row 40 vs. Row 9	46,150	8819,422	<0,001	Yes
Row 40 vs. Row 66	46,150	8819,422	<0,001	Yes
Row 40 vs. Row 23	44,370	8818,673	<0,001	Yes
Row 40 vs. Row 3	43,970	8818,505	<0,001	Yes
Row 40 vs. Row 71	43,970	8818,505	<0,001	Yes

Row 40 vs. Row 50	43,170	8818,168	<0,001	Yes
Row 40 vs. Row 67	42,470	8817,873	<0,001	Yes
Row 40 vs. Row 16	42,420	8817,852	<0,001	Yes
Row 40 vs. Row 24	42,420	8817,852	<0,001	Yes
Row 40 vs. Row 46	41,570	8817,495	<0,001	Yes
Row 40 vs. Row 55	40,770	8817,158	<0,001	Yes
Row 40 vs. Row 36	40,770	8817,158	<0,001	Yes
Row 40 vs. Row 72	40,770	8817,158	<0,001	Yes
Row 40 vs. Row 80	40,770	8817,158	<0,001	Yes
Row 40 vs. Row 79	40,570	8817,074	<0,001	Yes
Row 40 vs. Row 88	39,120	8816,464	<0,001	Yes
Row 40 vs. Row 18	38,460	8816,186	<0,001	Yes
Row 40 vs. Row 43	37,420	8815,748	<0,001	Yes
Row 40 vs. Row 75	35,770	8815,054	<0,001	Yes
Row 40 vs. Row 35	35,170	8814,801	<0,001	Yes
Row 40 vs. Row 1	34,620	8814,570	<0,001	Yes
Row 40 vs. Row 84	34,070	8814,338	<0,001	Yes
Row 40 vs. Row 4	32,420	8813,644	<0,001	Yes
Row 40 vs. Row 20	30,770	8812,950	<0,001	Yes
Row 40 vs. Row 76	29,120	8812,255	<0,001	Yes
Row 40 vs. Row 7	28,970	8812,192	<0,001	Yes
Row 40 vs. Row 83	27,670	8811,645	<0,001	Yes
Row 40 vs. Row 28	27,420	8811,540	<0,001	Yes
Row 40 vs. Row 27	26,270	8811,056	<0,001	Yes
Row 40 vs. Row 68	25,770	8810,845	<0,001	Yes
Row 40 vs. Row 32	25,270	8810,635	<0,001	Yes
Row 40 vs. Row 26	25,000	8810,521	<0,001	Yes
Row 40 vs. Row 59	24,120	8810,151	<0,001	Yes
Row 40 vs. Row 51	24,120	8810,151	<0,001	Yes
Row 40 vs. Row 54	22,570	88 9,499	<0,001	Yes
Row 40 vs. Row 47	22,420	88 9,435	<0,001	Yes
Row 40 vs. Row 57	19,230	88 8,093	<0,001	Yes
Row 40 vs. Row 56	17,840	88 7,508	<0,001	Yes
Row 40 vs. Row 42	17,470	88 7,352	<0,001	Yes
Row 40 vs. Row 63	14,120	88 5,942	0,059	No
Row 40 vs. Row 41	3,850	88 1,620	1,000	Do Not Test
Row 41 vs. Row 22	76,620	8832,245	<0,001	Yes
Row 41 vs. Row 14	73,420	8830,899	<0,001	Yes
Row 41 vs. Row 30	72,620	8830,562	<0,001	Yes
Row 41 vs. Row 53	70,420	8829,636	<0,001	Yes
Row 41 vs. Row 78	70,020	8829,468	<0,001	Yes
Row 41 vs. Row 87	69,920	8829,426	<0,001	Yes
Row 41 vs. Row 13	69,820	8829,384	<0,001	Yes
Row 41 vs. Row 37	69,420	8829,215	<0,001	Yes
Row 41 vs. Row 5	69,020	8829,047	<0,001	Yes
Row 41 vs. Row 60	66,620	8828,037	<0,001	Yes
Row 41 vs. Row 38	65,920	8827,742	<0,001	Yes
Row 41 vs. Row 29	65,820	8827,700	<0,001	Yes
Row 41 vs. Row 85	65,620	8827,616	<0,001	Yes
Row 41 vs. Row 6	65,120	8827,406	<0,001	Yes
Row 41 vs. Row 52	62,620	8826,354	<0,001	Yes
Row 41 vs. Row 69	62,520	8826,311	<0,001	Yes
Row 41 vs. Row 77	62,120	8826,143	<0,001	Yes
Row 41 vs. Row 61	61,620	8825,933	<0,001	Yes
Row 41 vs. Row 70	61,520	8825,891	<0,001	Yes
Row 41 vs. Row 86	60,420	8825,428	<0,001	Yes
Row 41 vs. Row 44	58,420	8824,586	<0,001	Yes
Row 41 vs. Row 58	57,520	8824,207	<0,001	Yes
Row 41 vs. Row 39	56,920	8823,955	<0,001	Yes
Row 41 vs. Row 31	55,020	8823,155	<0,001	Yes
Row 41 vs. Row 45	54,720	8823,029	<0,001	Yes
Row 41 vs. Row 21	53,720	8822,608	<0,001	Yes
Row 41 vs. Row 15	48,520	8820,420	<0,001	Yes
Row 41 vs. Row 11	47,720	8820,083	<0,001	Yes
Row 41 vs. Row 64	47,320	8819,915	<0,001	Yes
Row 41 vs. Row 10	46,150	8819,422	<0,001	Yes

Row 41 vs. Row 49	46,150	8819,422	<0,001	Yes
Row 41 vs. Row 2	46,150	8819,422	<0,001	Yes
Row 41 vs. Row 12	45,270	8819,052	<0,001	Yes
Row 41 vs. Row 8	43,570	8818,336	<0,001	Yes
Row 41 vs. Row 19	43,320	8818,231	<0,001	Yes
Row 41 vs. Row 62	42,520	8817,894	<0,001	Yes
Row 41 vs. Row 9	42,300	8817,802	<0,001	Yes
Row 41 vs. Row 66	42,300	8817,802	<0,001	Yes
Row 41 vs. Row 23	40,520	8817,053	<0,001	Yes
Row 41 vs. Row 3	40,120	8816,884	<0,001	Yes
Row 41 vs. Row 71	40,120	8816,884	<0,001	Yes
Row 41 vs. Row 50	39,320	8816,548	<0,001	Yes
Row 41 vs. Row 67	38,620	8816,253	<0,001	Yes
Row 41 vs. Row 16	38,570	8816,232	<0,001	Yes
Row 41 vs. Row 24	38,570	8816,232	<0,001	Yes
Row 41 vs. Row 46	37,720	8815,874	<0,001	Yes
Row 41 vs. Row 55	36,920	8815,538	<0,001	Yes
Row 41 vs. Row 36	36,920	8815,538	<0,001	Yes
Row 41 vs. Row 72	36,920	8815,538	<0,001	Yes
Row 41 vs. Row 80	36,920	8815,538	<0,001	Yes
Row 41 vs. Row 79	36,720	8815,454	<0,001	Yes
Row 41 vs. Row 88	35,270	8814,843	<0,001	Yes
Row 41 vs. Row 18	34,610	8814,566	<0,001	Yes
Row 41 vs. Row 43	33,570	8814,128	<0,001	Yes
Row 41 vs. Row 75	31,920	8813,433	<0,001	Yes
Row 41 vs. Row 35	31,320	8813,181	<0,001	Yes
Row 41 vs. Row 1	30,770	8812,950	<0,001	Yes
Row 41 vs. Row 84	30,220	8812,718	<0,001	Yes
Row 41 vs. Row 4	28,570	8812,024	<0,001	Yes
Row 41 vs. Row 20	26,920	8811,329	<0,001	Yes
Row 41 vs. Row 76	25,270	8810,635	<0,001	Yes
Row 41 vs. Row 7	25,120	8810,572	<0,001	Yes
Row 41 vs. Row 83	23,820	8810,025	<0,001	Yes
Row 41 vs. Row 28	23,570	88 9,919	<0,001	Yes
Row 41 vs. Row 27	22,420	88 9,435	<0,001	Yes
Row 41 vs. Row 68	21,920	88 9,225	<0,001	Yes
Row 41 vs. Row 32	21,420	88 9,015	<0,001	Yes
Row 41 vs. Row 26	21,150	88 8,901	<0,001	Yes
Row 41 vs. Row 59	20,270	88 8,531	<0,001	Yes
Row 41 vs. Row 51	20,270	88 8,531	<0,001	Yes
Row 41 vs. Row 54	18,720	88 7,878	<0,001	Yes
Row 41 vs. Row 47	18,570	88 7,815	<0,001	Yes
Row 41 vs. Row 57	15,380	88 6,473	0,013	Yes
Row 41 vs. Row 56	13,990	88 5,888	0,068	No
Row 41 vs. Row 42	13,620	88 5,732	0,100	Do Not Test
Row 41 vs. Row 63	10,270	88 4,322	0,840	Do Not Test

Table S4: Statistical tables for one way ANOVA using Sigma Plot 14.0. ANOVA table for data in figure 2.

**Source of Variation**	**DF**	**SS**	**MS**	**F**	**P**
Between Groups	7	16837,409	2405,344	439,032	<0,001
Residual	16	87,660	5,479		
Total	23	16925,069			

The differences in the mean values among the treatment groups are greater than would be expected by chance; there is a statistically significant difference (P = <0,001).

All Pairwise Multiple Comparison Procedures (Tukey Test):

Comparisons for factor:

**Comparison**	**Diff of Means**	**p q**	**P**	**P<0,050**
Row 4 vs. Row 6	63,100	846,693	<0,001	Yes
Row 4 vs. Row 8	62,500	846,249	<0,001	Yes
Row 4 vs. Row 3	55,960	841,409	<0,001	Yes
Row 4 vs. Row 7	39,590	829,296	<0,001	Yes
Row 4 vs. Row 5	6,250	8 4,625	0,071	No

Row 4 vs. Row 2	6,250	8 4,625	0,071	Do Not Test
Row 4 vs. Row 1	4,170	8 3,086	0,410	Do Not Test
Row 1 vs. Row 6	58,930	843,607	<0,001	Yes
Row 1 vs. Row 8	58,330	843,163	<0,001	Yes
Row 1 vs. Row 3	51,790	838,324	<0,001	Yes
Row 1 vs. Row 7	35,420	826,210	<0,001	Yes
Row 1 vs. Row 5	2,080	8 1,539	0,950	Do Not Test
Row 1 vs. Row 2	2,080	8 1,539	0,950	Do Not Test
Row 2 vs. Row 6	56,850	842,068	<0,001	Yes
Row 2 vs. Row 8	56,250	841,624	<0,001	Yes
Row 2 vs. Row 3	49,710	836,784	<0,001	Yes
Row 2 vs. Row 7	33,340	824,671	<0,001	Yes
Row 2 vs. Row 5	0,000	8 0,000	1,000	Do Not Test
Row 5 vs. Row 6	56,850	842,068	<0,001	Yes
Row 5 vs. Row 8	56,250	841,624	<0,001	Yes
Row 5 vs. Row 3	49,710	836,784	<0,001	Yes
Row 5 vs. Row 7	33,340	824,671	<0,001	Yes
Row 7 vs. Row 6	23,510	817,397	<0,001	Yes
Row 7 vs. Row 8	22,910	816,953	<0,001	Yes
Row 7 vs. Row 3	16,370	812,113	<0,001	Yes
Row 3 vs. Row 6	7,140	8 5,283	0,030	Yes
Row 3 vs. Row 8	6,540	8 4,839	0,054	No
Row 8 vs. Row 6	0,600	8 0,444	1,000	No

Table S5: Statistical tables for one way ANOVA using Sigma Plot 14.0. ANOVA table for data in figure 3.

Source of Variation	DF	SS	MS	F	P
Between Groups	8	14544,360	1818,045	190,106	<0,001
Residual	18	172,140	9,563		
Total	26	14716,500			

The differences in the mean values among the treatment groups are greater than would be expected by chance; there is a statistically significant difference (P = <0,001).

All Pairwise Multiple Comparison Procedures (Tukey Test):

Comparisons for factor:

Comparison	Diff of Means	p q	P	P<0,050
Row 5 vs. Row 9	67,950	938,058	<0,001	Yes
Row 5 vs. Row 2	67,950	938,058	<0,001	Yes
Row 5 vs. Row 4	62,600	935,061	<0,001	Yes
Row 5 vs. Row 7	58,350	932,681	<0,001	Yes
Row 5 vs. Row 1	53,050	929,713	<0,001	Yes
Row 5 vs. Row 3	32,350	918,119	<0,001	Yes
Row 5 vs. Row 8	21,600	912,098	<0,001	Yes
Row 5 vs. Row 6	21,500	912,042	<0,001	Yes
Row 6 vs. Row 9	46,450	926,016	<0,001	Yes
Row 6 vs. Row 2	46,450	926,016	<0,001	Yes
Row 6 vs. Row 4	41,100	923,020	<0,001	Yes
Row 6 vs. Row 7	36,850	920,639	<0,001	Yes
Row 6 vs. Row 1	31,550	917,671	<0,001	Yes
Row 6 vs. Row 3	10,850	9 6,077	0,010	Yes
Row 6 vs. Row 8	0,1000	90,0560	1,000	No
Row 8 vs. Row 9	46,350	925,960	<0,001	Yes
Row 8 vs. Row 2	46,350	925,960	<0,001	Yes
Row 8 vs. Row 4	41,000	922,964	<0,001	Yes
Row 8 vs. Row 7	36,750	920,583	<0,001	Yes
Row 8 vs. Row 1	31,450	917,615	<0,001	Yes
Row 8 vs. Row 3	10,750	9 6,021	0,011	Yes
Row 3 vs. Row 9	35,600	919,939	<0,001	Yes
Row 3 vs. Row 2	35,600	919,939	<0,001	Yes
Row 3 vs. Row 4	30,250	916,943	<0,001	Yes
Row 3 vs. Row 7	26,000	914,562	<0,001	Yes
Row 3 vs. Row 1	20,700	911,594	<0,001	Yes
Row 1 vs. Row 9	14,900	9 8,345	<0,001	Yes
Row 1 vs. Row 2	14,900	9 8,345	<0,001	Yes

Row 1 vs. Row 4	9,550	9	5,349	0,029	Yes
Row 1 vs. Row 7	5,300	9	2,968	0,503	No
Row 7 vs. Row 9	9,600	9	5,377	0,028	Yes
Row 7 vs. Row 2	9,600	9	5,377	0,028	Yes
Row 7 vs. Row 4	4,250	9	2,380	0,749	No
Row 4 vs. Row 9	5,350	9	2,996	0,491	No
Row 4 vs. Row 2	5,350	9	2,996	0,491	Do Not Test
Row 2 vs. Row 9	0,000	9	0,000	1,000	Do Not Test
